# Involvement of HemI, an ECF sigma factor, in hemin acquisition and antibiotic susceptibility in *Stenotrophomonas maltophilia*

**DOI:** 10.3389/fcimb.2025.1722701

**Published:** 2025-12-23

**Authors:** Chun-Hsing Liao, Ren-Hsuan Ku, Hsu-Feng Lu, En-Wei Hu, Li-Hua Li, Tsuey-Ching Yang

**Affiliations:** 1 Division of Infectious Disease, Far Eastern Memorial Hospital, New Taipei City, Taiwan, R.O.C; 2 Department of Medicine, National Yang Ming Chiao Tung University, Taipei, Taiwan, R.O.C; 3 Department of Biotechnology and Laboratory Science in Medicine, National Yang Ming Chiao Tung University, Taipei, Taiwan, R.O.C; 4 Department of Medical Laboratory Science and Biotechnology, Asia University, Taichung, Taiwan, R.O.C; 5 Department of Pathology and Laboratory Medicine, Taipei Veterans General Hospital, Taipei, Taiwan, R.O.C; 6 School of Medical Laboratory Science and Biotechnology, College of Medical Science and Technology, Taipei Medical University, Taipei, Taiwan, R.O.C

**Keywords:** *Stenotrophomonas maltophilia*, sigma factor, HemI, HemR, hemin acquisition, surface signaling cascade

## Abstract

**Background:**

Hemin is a major source of iron for pathogens in infectious niches. The FecIRA-like surface signaling cascade is a common regulatory system for iron acquisition by pathogens. This system consists of a FecA-like TonB-dependent transporter (TBDT), a FecR-like inner membrane anti-sigma factor, and a FecI-like extracytoplasmic function (ECF) sigma factor. Beyond iron acquisition, FecIRA-like systems have been reported to regulate additional physiological processes. The known hemin acquisition system in *Stenotrophomonas maltophilia* includes HemA, a TBDT; HemU, an inner membrane transporter; and the TonB1–ExbB1–ExbD1a–ExbD1b complex, a multi-subunit motor that energizes HemA. Fur and HemP are the primary regulators involved in hemin utilization. In this study, we identified a novel FecIRA-like regulatory system, i.e., HemI–HemR–HemA_D_.

**Methods:**

The regulatory role of HemI was examined using promoter–*xylE* transcriptional fusion constructs and real-time PCR. Mutants associated with the *hemI–hemR–hemA_D_* operon were generated and evaluated for iron utilization, swimming motility, oxidative stress tolerance, and antibiotic susceptibility.

**Results:**

The *hemI–hemR–hemA_D_* operon was repressed by Fur–Fe^2+^ under iron-replete conditions. Its expression was partially derepressed under iron depletion and further derepressed in the presence of hemin; however, the operon showed no autoregulation. HemI was essential for hemin acquisition. The overexpression of *hemI* in the *S. maltophilia* KJ strain increased the susceptibility to levofloxacin (LVX) and trimethoprim–sulfamethoxazole (SXT). All *S. maltophilia* isolates examined displayed increased minimum inhibitory concentrations (MICs) for ceftazidime (CAZ) and minocycline (MIN) under the iron-depleted and hemin-available conditions; notably, the changes in the MICs of LVX and SXT were strain-dependent.

**Conclusion:**

HemI, a novel ECF sigma factor, not only regulates hemin acquisition but also contributes to antibiotic susceptibility under iron-limited and hemin-available conditions.

## 1 Introduction

Iron is indispensable to bacteria because it participates in numerous essential biological processes such as DNA replication, transcription, and energy generation. However, under aerobic conditions, excess iron is toxic: ferrous iron catalyzes the Fenton reaction, producing hydroxyl radicals from hydrogen peroxide (Fenton, 1894). Owing to this dual nature—essential yet potentially lethal—iron acquisition must be tightly regulated. Both iron depletion and the presence of specific iron sources induce the expression of iron uptake systems (Sheldon et al., 2016). In Gram-negative bacteria, several regulatory mechanisms have been described for controlling these systems, including the Fur transcriptional repressor, the transcription factor HemP, and the FecIRA-like surface signaling cascade (SSC).

Fur is a global Fe^2+^-dependent transcriptional repressor conserved across bacteria. When intracellular iron is sufficient, Fur forms a complex with Fe^2+^ that binds to a conserved DNA sequence known as the Fur box, located near Fur-regulated promoters, thereby repressing transcription (Hantke, 1981). Under iron limitation, Fur cannot bind its corepressor, leading to the derepression of Fur-regulated genes (Andrews et al., 2003; Troxell and Hassan, 2013).

HemP (also known as HmuP) regulates hemin acquisition in various bacteria, including *Yersinia enterocolitica*, *Ensifer meliloti*, *Bradyrhizobium japonicum*, *Burkholderia multivorans*, and *Stenotrophomonas maltophilia* (Amarelle et al., 2010; Escamilla-Hernandez and O’Brian, 2012; Stojiljkovic and Hantke, 1992). HemP is crucial for the expression of the TonB-dependent transporters (TBDTs) responsible for hemin uptake in *Y. enterocolitica*, *E. meliloti*, *B. japonicum*, and *B. multivorans* (Amarelle et al., 2010; Escamilla-Hernandez and O’Brian, 2012; Sato et al., 2017; Stojiljkovic and Hantke, 1992). Interestingly, in *S. maltophilia*, HemP negatively regulates *hemA* under iron-depleted conditions (Shih et al., 2022).

The role of SSCs in iron acquisition was first described in the FecI–FecR–FecA system of *Escherichia coli*, in which it regulates the uptake of ferric citrate (Visca et al., 2002). Since then, FecIRA-like systems have been identified in many bacteria, including *fecIRA*, *hasISR*, and *hxuIRA* in *Pseudomonas aeruginosa*; *pupIRB* in *Pseudomonas putida*; *hurIR-bhuR* in *Bordetella pertussis*; *rhuIR-bhuR* in *Bordetella avium*; and *prhIRA* in *Ralstonia solanacearum* (Brickman et al., 2006; Brito et al., 2002; Smith and Wilks, 2015; Kirby et al., 2004; King et al., 2005; Koster et al., 1994). A typical FecIRA-like SSC consists of a FecA-like TBDT that senses extracellular stimuli and transports the iron complex, a FecI-like extracytoplasmic function (ECF) sigma factor, and a FecR-like inner membrane anti-sigma factor that transduces the signal from the periplasm to the cytoplasm (Braun and Mahren, 2005). Upon ligand binding, the TBDT initiates a signal cascade that releases the sigma factor from the anti-sigma factor, allowing transcriptional activation. In general, these three genes are organized as an operon (Braun et al., 2006), although the *fecIRA* genes in *E. coli* are an exception (Härle et al., 1995). Beyond iron uptake, FecIRA-like systems also regulate virulence, fitness *in vivo*, and oxidative stress responses (Brito et al., 2002; Cai et al., 2021; Otero-Asman et al., 2019).

To limit pathogen growth, host organisms sequester iron through “nutritional immunity” using high-affinity iron- and heme-binding proteins such as hemoglobin, hemopexin, haptoglobin, ferritin, and transferrin (Murdoch and Skaar, 2022). To overcome this barrier, pathogens have evolved diverse iron acquisition systems specialized for different iron sources (Braun and Killman, 1999). Because the majority of host iron is bound in heme, hemin acquisition is crucial for pathogen survival.

*Stenotrophomonas maltophilia* is a ubiquitous environmental bacterium and an emerging multidrug-resistant nosocomial pathogen, primarily affecting cystic fibrosis patients and immunocompromised individuals (Brooke, 2012). To thrive in various environments and infection sites, *S. maltophilia* possesses multiple iron acquisition systems. Similarly to the majority of bacteria, it produces the siderophore stenobactin (Nas and Cianciotto, 2017; Yeh et al., 2025). Additional systems include FciTABC–FeoABI for ferric citrate uptake, PacIRA for xenosiderophore utilization, and the HemA/HemU/TonB1 system for hemin acquisition (Liao et al., 2024, 2022; Pan et al., 2022). Known regulatory factors include Fur, AmpR, and HemP (Kalidasan et al., 2018; Liao et al., 2020; Shih et al., 2022). Here, we identify a previously uncharacterized FecIRA-like system, i.e., HemI–HemR–HemA_D_, that regulates both the hemin acquisition and antibiotic susceptibility in *S. maltophilia*.

## 2 Materials and methods

### 2.1 Bacterial strains, plasmids, and primers

The bacterial strains and plasmids used in this study are listed in **Supplementary Table S1**. The primers are listed in **Supplementary Table S2**.

### 2.2 Construction of in-frame deletion mutants

In-frame, unmarked deletion mutants were generated by double-crossover homologous recombination as described previously (Yang et al., 2009). Two DNA fragments (373 bp upstream and 331 bp downstream of *hemI*) were amplified by PCR using KJ genomic DNA as the template and the primer pairs HemIN-F/R and HemIC-F/R (**Supplementary Table S2**) and then cloned into the pEX18Tc vector to construct pΔHemI (**Supplementary Table S1**). DNA segments containing full-length *hemR*, *hemA_D_*, and *smlt3897* were amplified using primers HemR-F/R, HemA_D_-F/R, and 3897-F/R (**Supplementary Table S2**) and then cloned into pEX18Tc to generate pHemR, pHemA_D_, and p3897. The internal PstI–PstI fragments of these plasmids were excised by *Pst*I restriction digestion and self-ligation to yield pΔHemR, pΔHemA_D_, and pΔ3897 (**Supplementary Table S1**). The pEX18Tc-derived plasmids were introduced into *S. maltophilia* KJ or KJΔEnt (Liao et al., 2020) by conjugation. Transconjugants were selected on Luria–Bertani (LB) agar containing 1.5 μg/ml norfloxacin and 30 μg/ml tetracycline, followed by counter-selection on 10% sucrose to isolate the deletion mutants. Mutants were confirmed by PCR and PCR amplicon sequencing. Double and triple mutants were constructed sequentially from single mutants using the same procedure.

### 2.3 Construction of complementation plasmids pHemI

The *hemI* gene was amplified from KJ genomic DNA using primers HemI-F/R (**Supplementary Table S2**). The PCR amplicon was digested with *Hin*dIII and *Eco*RI and then cloned into pRK415 to generate pHemI (**Supplementary Table S1**). The *hemI* gene was inserted in the correct orientation to be transcribed by the *lacZ* promoter of pRK415.

### 2.4 Hemin utilization assay

As previously reported, *S. maltophilia* KJ exhibits poor growth in LB supplemented with 50 μg/ml 2,2′-dipyridyl (DIP), unless an external iron source is provided (Liao et al., 2020). Log-phase cultures were adjusted to 2 × 10^5^ colony-forming units (CFU)/μl, serially diluted 10-fold, and 5 µl of each dilution was spotted onto LB agar with or without the indicated additives. The plates were incubated at 37°C for 24 h, and growth was monitored by photography. Experiments were performed in triplicate.

### 2.5 Construction of promoter–*xylE* transcriptional fusions

DNA fragments containing the promoter regions of *hemI* (373 bp) and *tonB1* (444 bp) were amplified by PCR using KJ genomic DNA and the primer sets HemIN-F/R and TonB1N-F/R (**Supplementary Table S2**). The 373- and 444-bp PCR amplicons were cloned into pXylE (Chen et al., 2011) to yield pHemI_xylE_ and pTonB1_xylE_, respectively (**Supplementary Table S1**).

### 2.6 Catechol 2,3-dioxygenase activity assay

Catechol 2,3-dioxygenase (C23O), which is encoded by *xylE*, converts catechol to 2-hydroxymuconic semialdehyde, which was quantified spectrophotometrically at 375 nm (Lin et al., 2009). One unit of activity (Uc) was defined as the amount of enzyme converting 1 nmol of catechol per minute. Specific activity was expressed as Uc per OD_450 nm_ (optical density at 450 nm) unit of cells. In our previous study, we have established that OD_450nm_ is more sensitive than OD_600 nm_ for monitoring the growth of *S. maltophilia* KJ. An OD_450 nm_ of 1 for *S. maltophilia* KJ corresponds to 3.6 × 10^8^ cells/ml (Lin et al., 2009). Data represent three independent experiments.

### 2.7 Real-time PCR

RNA was extracted from log-phase cells using the HiYield™ Total RNA Extraction Kit *Mini* (Arrowtec Life Science, New Taipei City, Taiwan) and treated with RNase-free DNase I (Arrowtec Life Science). DNA-free RNA was reverse-transcribed using the High-Capacity cDNA Reverse Transcription Kit (Applied Biosystems, Waltham, MA, USA) according to the manufacturer’s protocol. Real-time PCR was performed using SYBR qPCR Master Mix (Arrowtec Life Science) on an ABI StepOnePlus™ system. The primers are listed in **Supplementary Table S2**. Relative expression was calculated using the ΔΔ*C*_T_ method (Livak and Schmittgen, 2001) with 16S rRNA as the internal control. Each experiment was performed in triplicate.

### 2.8 Beta-lactamase activity assay

Overnight cultures were inoculated into fresh LB to an OD_450 nm_ of 0.15. After 3 h at 37°C, ceftazidime (50 µg/ml) was added for 30 min. Whole-cell β-lactamase activity was measured by nitrocefin hydrolysis (Lin et al., 2009). Specific activity (Un/mg) was defined as nanomoles of nitrocefin hydrolyzed per minute per milligram of protein. The protein concentrations were determined using Bio-Rad reagent with bovine serum albumin (BSA) as the standard.

### 2.9 Antibiotic susceptibility testing

Susceptibility to ceftazidime, levofloxacin, trimethoprim–sulfamethoxazole, and minocycline was determined with the E-test (Liofilchem, Roseto degli Abruzzi, Italy) following the manufacturer’s guidelines. The minimum inhibitory concentrations (MICs) were read at the intersection of the inhibition ellipse with the strip.

### 2.10 Statistical analysis

All data are presented as the mean ± standard deviation (SD). The mean and SD were obtained from three independent experiments. Student’s *t*-test was used for pairwise comparisons as indicated, considering a *p*-value of 0.05 or less as significant.

## 3 Results

### 3.1 HemI, an ECF sigma factor, is involved in hemin utilization

**Figure 1:**
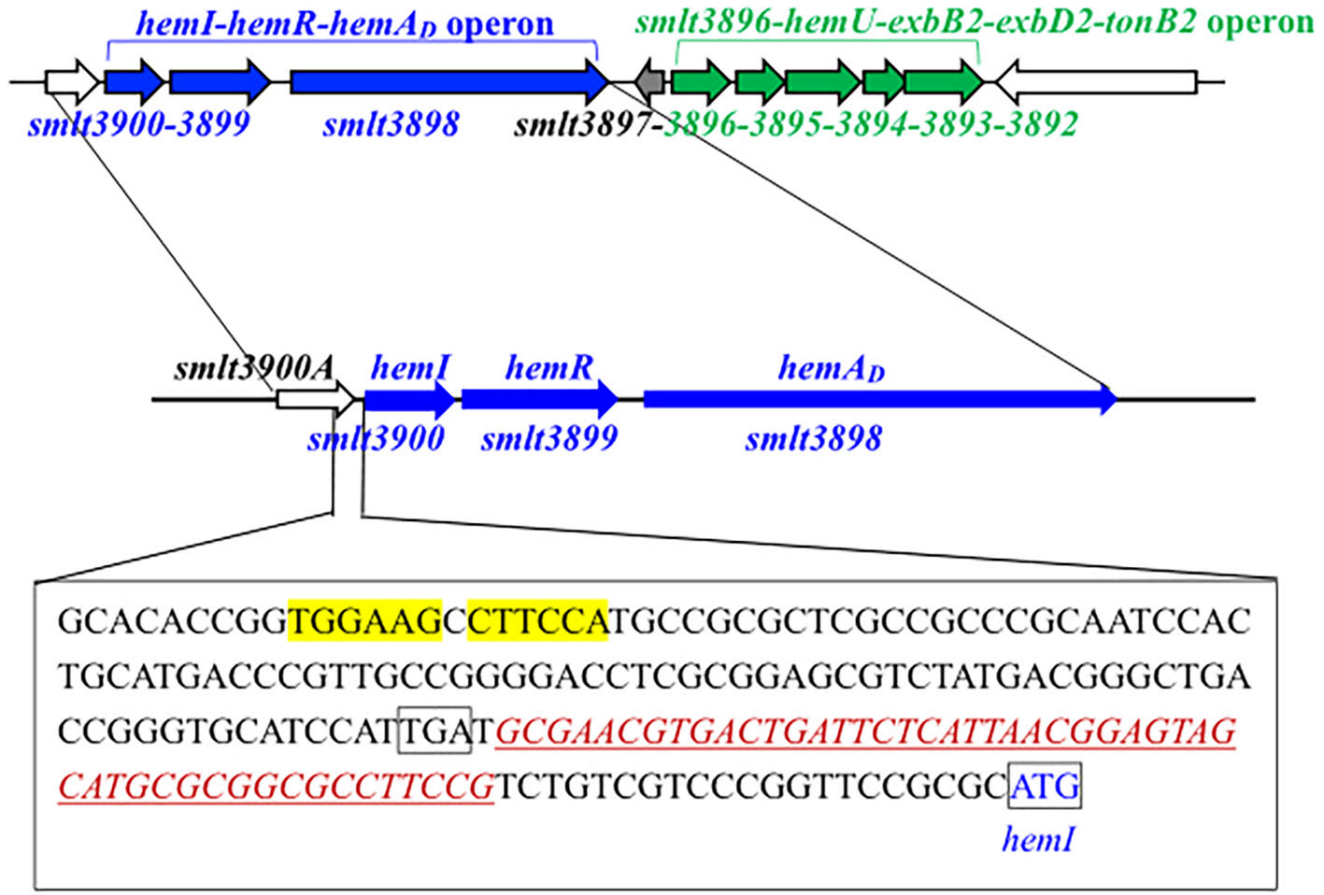
Genetic organization and predicted Fur box of the *hemI*–*hemR*–*hemA_D_* operon in *Stenotrophomonas maltophilia*. Arrows indicate gene orientation. The DNA sequence spanning the 3′-end of *smlt3900A* and the intergenic region between *smlt3900A* and *hemI* is shown in the rectangle. The predicted promoter is underlined (predicted via https://www.fruitfly.org/seq_tools/promoter.html). The putative Fur box (per García et al., 2015) is highlighted in yellow.

A link between the *smlt3896*–*hemU*–*exbB2*–*exbD2*–*tonB2* operon and hemin acquisition in *S. maltophilia* was revealed in our previous study (Liao et al., 2024). A four-gene cluster (*smlt3897*–*smlt3900*) located upstream of this operon drew our attention (**Figure 1**). *smlt3897*, which was divergently transcribed relative to the *smlt3896*–*hemU*–*exbB2*–*exbD2*–*tonB2* operon, encodes an 85-amino acid (aa) protein of unknown function. *smlt3900* encodes a putative TBDT. The protein encoded by *smlt3899* is an inner membrane FecR family protein with a transmembrane region. *smlt3898* encodes a 166-aa cytoplasmic protein with a conserved RpoE domain. The products of *smlt3900*, *smlt3899*, and *smlt3898* appeared to constitute a FecIRA-like SSC. Based on subsequent analyses, we annotated *smlt3900*, *smlt3899*, and *smlt3898* as *hemI*, *hemR*, and *hemA_D_*, respectively.

**Figure 2:**
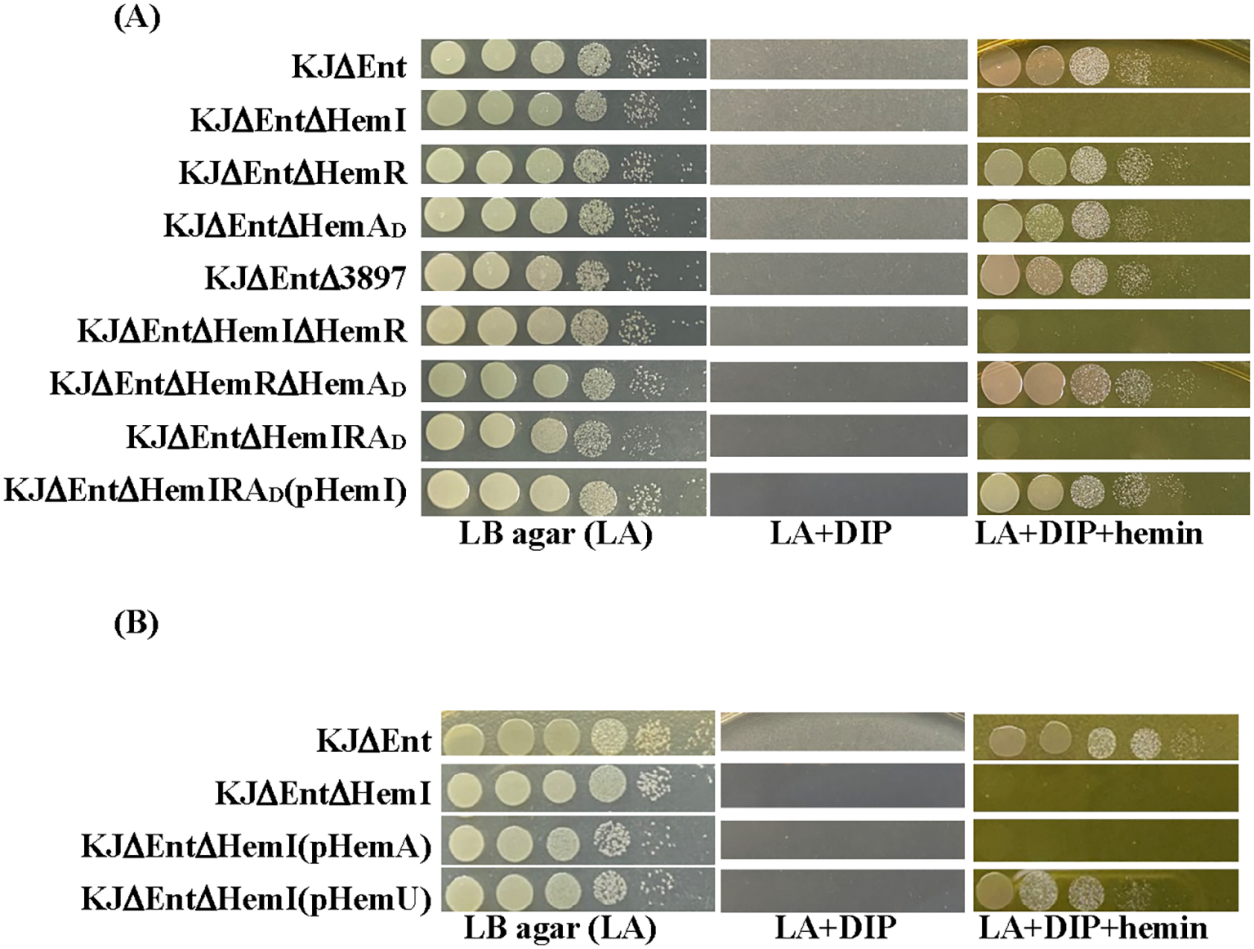
Cell viability of wild-type KJ and derived mutants under the iron-replete (LA), iron-depleted (LA + DIP), and iron-depleted plus hemin (LA + DIP + hemin) conditions. Bacteria (2 × 10^5^ CFU/μl) were serially diluted 10-fold and spotted onto agar for 24-h incubation. DIP, 50 μg/ml; hemin, 150 μM. **(A)** Roles of the *hemI*–*hemR*–*hemA_D_* operon and *smlt3897* in hemin utilization. **(B)** Regulatory role of HemI in *hemP*–*hemA*–*smlt0796*–*smlt0797* and *smlt3896*–*hemU*–*exbB2*–*exbD2*–*tonB2* operons.

Given this genomic organization (**Figure 1**), we hypothesized that the *hemI*–*hemR*–*hemA_D_* operon and *smlt3897* contribute to hemin acquisition. *S. maltophilia* KJ harbors the siderophore stenobactin, which mediates iron acquisition under iron-limited stress (Yeh et al., 2025). To avoid the confounding effects of stenobactin, we constructed in-frame deletion mutants of *smlt3897* and the *hemI*–*hemR*–*hemA_D_* operon in KJΔEnt, a stenobactin-null mutant (Liao et al., 2020), and assessed their ability to use hemin as the sole iron source under iron-depleted conditions. Among all mutants examined, KJΔEntΔHemI, KJΔEntΔHemIΔHemR, and KJΔEntΔHemIRA_D_ lost the ability to utilize hemin for growth, which was restored by *hemI* complementation (**Figure 2A**, **Supplementary Figure S1**), indicating that HemI is a key sigma factor required for hemin utilization. We further examined whether the *hemI*–*hemR*–*hemA_D_* operon contributed to the uptake of ferri-stenobactin or ferric citrate. No positive results were observed **(Supplementary Figure S2**).

Because HemI is required for hemin utilization, we speculated that HemA_D_ functions as the TBDT for hemin uptake. However, as shown in **Figure 2A**, this assumption was not supported. Sequence analysis of *hemA_D_* from strain KJ revealed that nucleotides 22–24 represent TGA, generating a premature stop codon and truncating HemA_D_ to 7 aa. We therefore used *smlt3898* from *S. maltophilia* K279a (Crossman et al., 2008) as a reference. The two alleles shared 97% DNA identity and encodes a protein of 98% identity when the premature stop codon was ignored (**Supplementary Figure S3**), indicating that HemA_D_ in strain KJ is a mutation-mediated truncated variant, herein designated HemA_D_ (D = defective). We also considered the possibility that the *hemA* gene of the KJ strain has an alternative start codon downstream the premature stop codon and generates a smaller TBDT protein. Nevertheless, the smaller TBDT, if present, was not involved in hemin uptake (**Figure 2A**).

### 3.2 Hemin acquisition-associated genes are differentially regulated by the growth phase and iron level and by hemin, Fur, HemP, and HemI

Integrating our previous findings (Liao et al., 2024; Shih et al., 2022) with the above results, we identified at least three structural components (i.e., HemA, HemU, and TonB1–ExbB1–ExbD1a–ExbD1b) and three regulators (i.e., Fur, HemP, and HemI) involved in hemin acquisition by *S. maltophilia*. These nine genes are distributed across five transcripts: *fur*, *hemP*–*hemA*–*smlt0796*–*smlt0797*, *smlt3896*–*hemU*–*exbB2*–*exbD2*–*tonB2*, *tonB1*–*exbB1*–*exbD1a*–*exbD1b*, and *hemI*–*hemR*–*hemA_D_* (**Supplementary Figure S4**).

As iron homeostasis genes are typically responsive to the iron levels and iron source availability (Braun, 2003), we sought to define how these four operons are transcriptionally regulated. Promoter–*xylE* fusions—pHemP_xylE_ (Shih et al., 2022), p3896_xylE_ (Liao et al., 2024), pHemI_xylE_, and pTonB1_xylE_—were constructed to measure the promoter activity.

**Figure 3:**
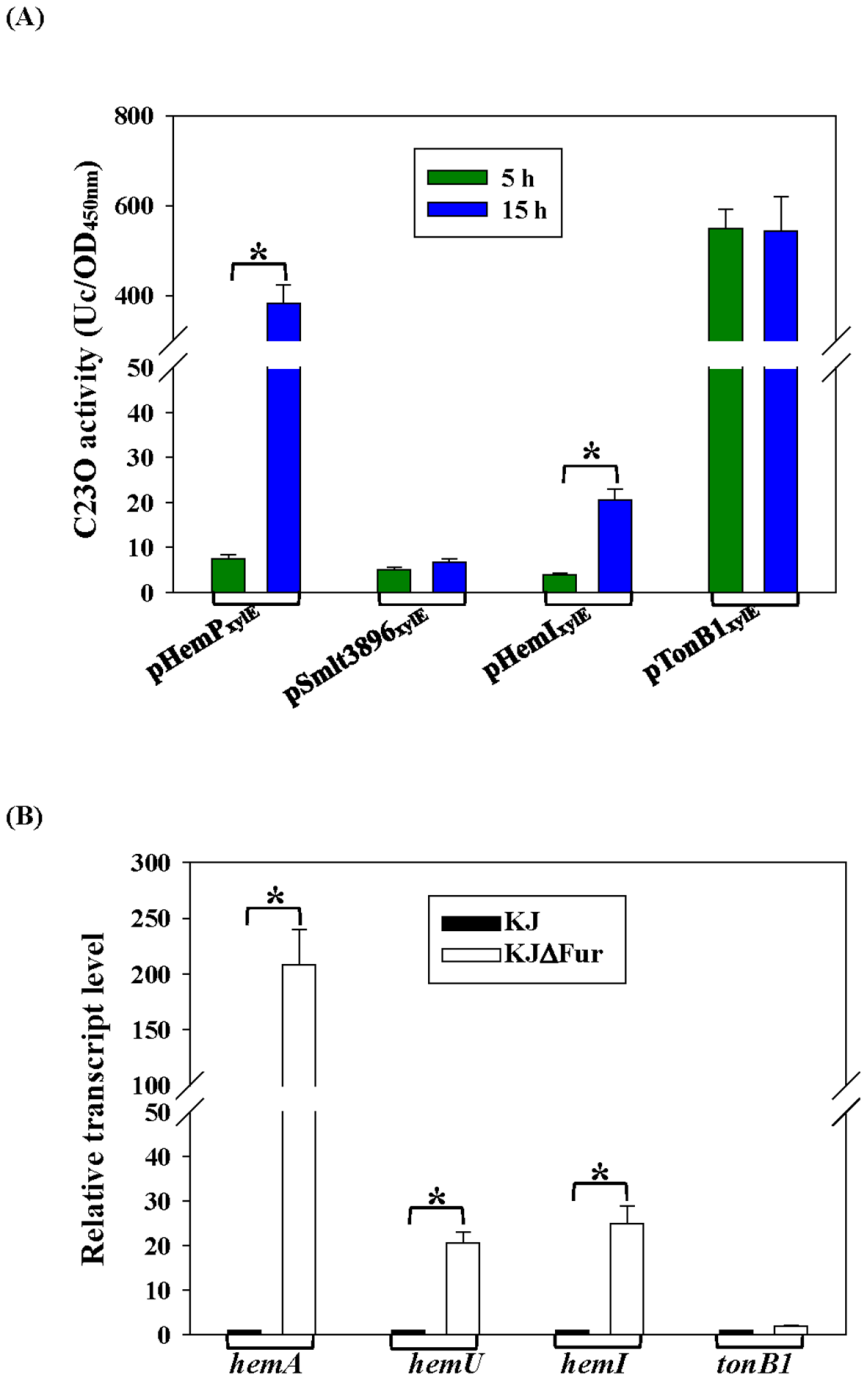
Effects of growth phase and Fur on the transcription of hemin acquisition-associated operons. Data represent the mean ± SD of three independent experiments. Statistical significance was determined using a two-tailed Student’s *t*-test (*p ≤ 0.05). **(A)** Impacts of growth phase on the transcription of hemin acquisition-associated operons. Plasmid (pHemP_xylE_, pSmlt3896_xylE_, pHemI_xylE_, or pTonB1_xylE_) was transported into KJ, and the 2,3-dioxygenase (C23O) activities expressed by these strains were determined. **(B)** Roles of Fur in the transcription of hemin acquisition-associated operons. The *hemA*, *hemU*, *hemI*, and *tonB1* transcripts of KJ and KJΔFur were determined using real-time PCR. The relative transcript levels were calculated using the transcript level of KJ cells as 1.

In the logarithmic phase, only *tonB1*–*exbB1*–*exbD1a*–*exbD1b* showed strong intrinsic expression (**Figure 3A**). *hemP*–*hemA*–*smlt0796*–*smlt0797* and *hemI*–*hemR*–*hemA_D_* were growth phase-regulated and were upregulated in the stationary phase, whereas *smlt3896*–*hemU*–*exbB2*–*exbD2*–*tonB2* remained weakly expressed under iron-replete conditions regardless of the growth phase (**Figure 3A**).

Fur is the global iron homeostasis repressor in Gram-negative bacteria. Real-time PCR comparing wild-type KJ and its fur-deletion mutant (KJΔFur) showed increased *hemA*, *hemU*, and *hemI* transcripts—but not *tonB1*—in KJΔFur (**Figure 3B**), indicating that *hemA*, *hemU*, and *hemI* belong to the Fur regulon. A putative Fur box (García et al., 2015) was identified upstream of *hemI* (**Figure 1**).

**Figure 4:**
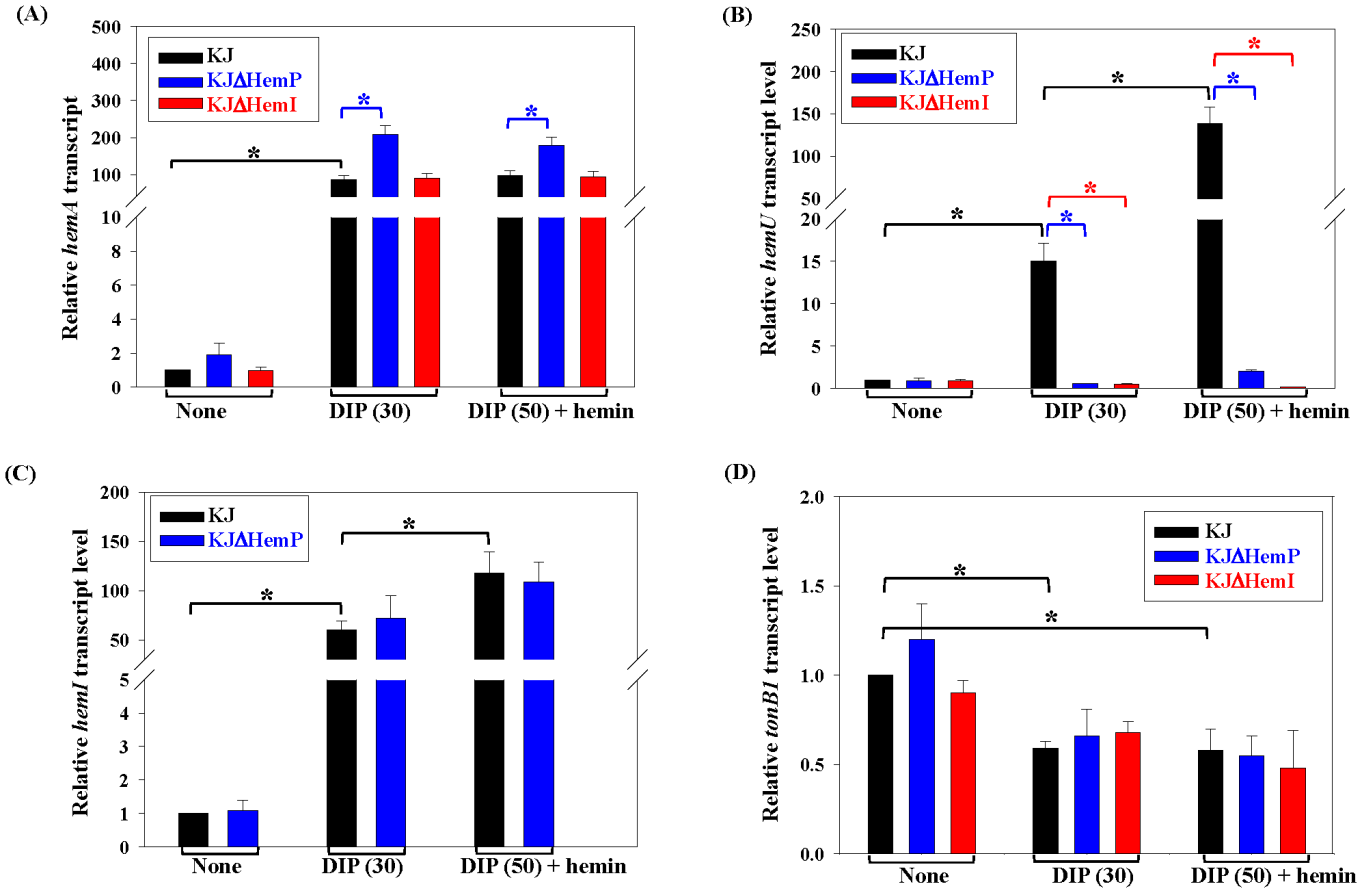
Effects of iron level, hemin availability, and the regulators HemP/HemI on the transcription of hemin acquisition-associated operons. Wild-type KJ, KJΔHemP, and KJΔHemI were cultured in the medium as indicated for 15 h The transcript levels of *hemA***(A)**, *hemU***(B)**, *hemI***(C)**, and *tonB1***(D)** were determined using real-time PCR and normalized to 16S rRNA. Fold change is shown relative to Luria–Bertani (LB)-grown KJ. Data are the mean ± SD of three independent experiments. Significance was determined using a two-tailed Student’s *t*-test (*p ≤ 0.05).

We next examined the effects of iron limitation and hemin supplementation and the roles of HemP and HemI in these regulatory circuits using real-time PCR. As reported earlier, the addition of 30 μg/ml DIP generates iron-depleted stress without impairing KJ growth (Liao et al., 2020), whereas 50 µg/ml DIP blocks growth unless an external iron source is provided. Thus, 30 μg/ml DIP was used to mimic iron depletion. The *hemA* transcript was upregulated under iron-depleted conditions; however, the addition of hemin did not further alter its expression. Within this iron depletion-mediated regulatory pathway, HemP functioned as a negative regulator, whereas HemI played no role (**Figure 4A**). Under iron-depleted conditions, the expression of *hemU* increased and was further enhanced by the presence of hemin; both HemP and HemI were required for this DIP- and hemin-responsive upregulation (**Figure 4B**). The expression of *hemI* itself was influenced by iron and hemin availability, but remained unaffected by HemP (**Figure 4C**). In contrast, the transcript levels of *tonB1* were moderately reduced in the DIP medium, independent of HemP and HemI (**Figure 4D**).

**Figure 5:**
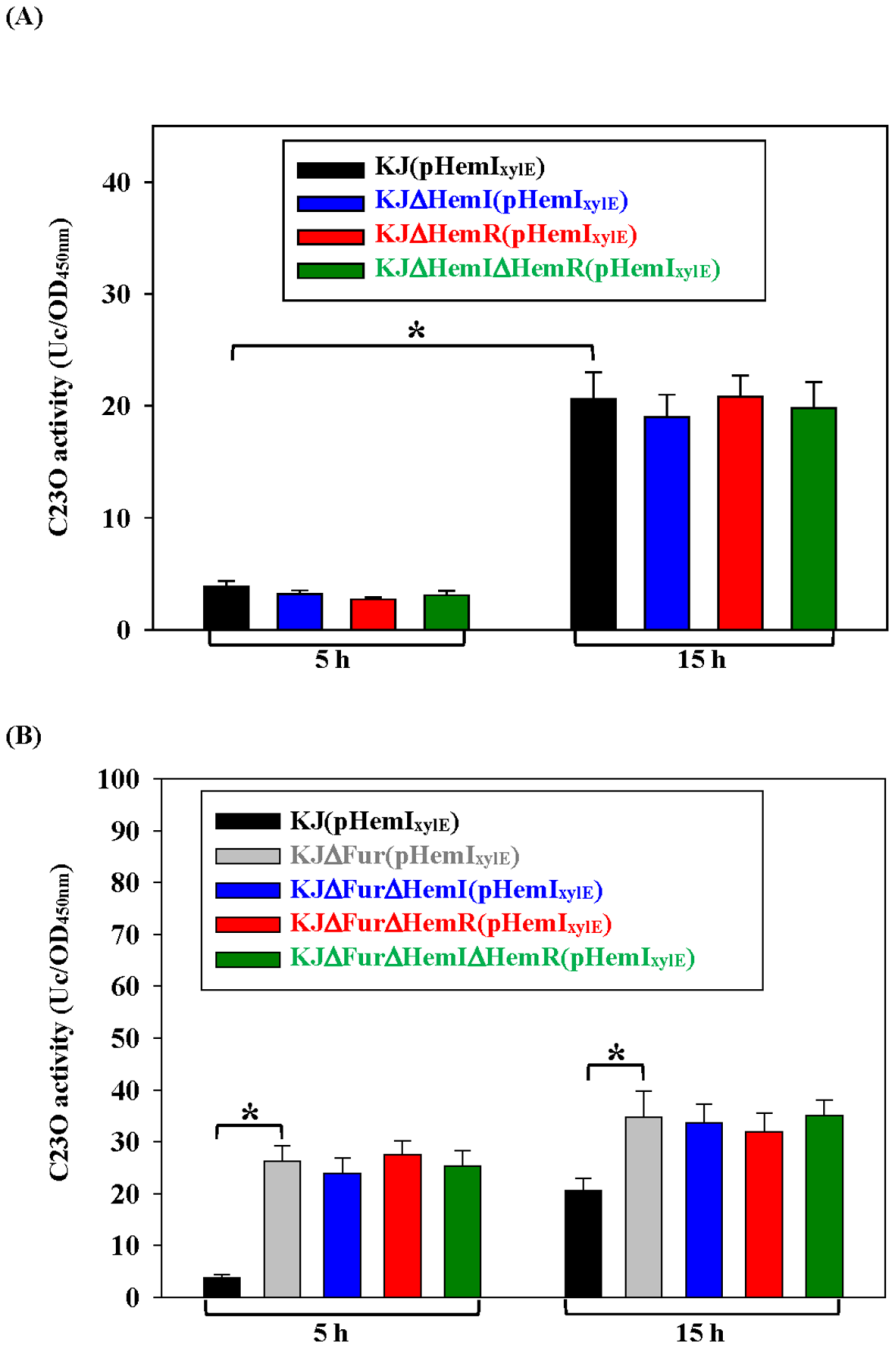
Autoregulation of the *hemI*–*hemR*–*hemA_D_* operon in *Stenotrophomonas maltophilia* KJ. Plasmid (pHemI_xylE_) was transported into KJ **(A)** or KJΔFur **(B)** and their derived mutants as indicated. The catechol 2,3-dioxygenase (C23O) activities expressed by these strains were determined. Data are the mean ± SD of three independent experiments. Significance was determined using a two-tailed Student’s *t*-test (*p ≤ 0.05).

Because many ECF sigma factors autoregulate their own operons, we examined whether *hemI*–*hemR*–*hemA_D_* is autoregulatory. In wild-type cells, the promoter activity increased in the stationary phase (**Figure 5A**), consistent with the data in **Figure 3A**. If HemR functions as an anti-sigma factor and HemI is autoregulated, the deletion of *hemR* should elevate the promoter activity, which should return to the baseline in a *ΔhemRΔhemI* double mutant. However, the deletion of *hemI*, *hemR*, or both had no effect on iron-replete LB (**Figure 5A**). Suspecting that Fur-mediated repression masked autoregulation, we repeated the assay in KJΔFur, where the operon was derepressed. The promoter activity increased in both log and stationary phases (**Figure 5B**), but remained unaffected by the additional *ΔhemI* and/or *ΔhemR* mutations (**Figure 5B**), confirming the absence of autoregulation.

### 3.3 Complementation of the *hemI* mutant with *hemU* restores hemin utilization

HemI proved essential for the expression of *smlt3896*–*hemU*–*exbB2*–*exbD2*–*tonB2* (**Figure 4B**), but had little impact on *hemP*–*hemA*–*smlt0796*–*smlt0797* (**Figure 4A**). Loss of *hemI* almost abolished the hemin-supported growth under iron-depleted conditions (**Figure 2A**). We hypothesized that this defect stemmed from insufficient HemU to support hemin utilization. To test this, we complemented KJΔEntΔHemI with pHemA or pHemU. Only KJΔEntΔHemI(pHemU) regained hemin-supported viability comparable to the parental KJΔEnt strain, whereas pHemA complementation failed (**Figure 2B**, **Supplementary Figure S5**).

### 3.4 HemI overexpression alters the antibiotic susceptibility

FecIRA-like SSC systems have been linked to functions beyond iron uptake (Brito et al., 2002; Cai et al., 2021; Otero-Asman et al., 2019). To determine whether HemI activation influences motility, oxidative stress tolerance, or antibiotic susceptibility, we overexpressed *hemI* in wild-type KJ. Overexpression had no effect on swimming or oxidative stress tolerance (**Supplementary Figure S6**). We next examined susceptibility to clinically relevant drugs—ceftazidime, levofloxacin, trimethoprim/sulfamethoxazole, and minocycline. *S. maltophilia* KJ is intrinsically ceftazidime-resistant (MIC > 256 μg/ml) due to inducible L1/L2 β-lactamases (Hu et al., 2008). Ceftazidime-induced β-lactamase activity was similar between KJ and KJ(pHemI) (**Supplementary Figure S7**), suggesting that HemI does not affect β-lactamase induction. We therefore used KJ2, an L1/L2-deficient KJ mutant (Chen et al., 2011), to assess β-lactam susceptibility. pHemI increased the ceftazidime MIC in KJ2 from 0.19 to 0.25 μg/ml (**Table 1**), indicating that HemI contributes to non-β-lactamase-mediated β-lactam resistance. HemI overexpression also increased the susceptibility to levofloxacin and trimethoprim/sulfamethoxazole, but left the minocycline susceptibility unchanged (**Table 1**).

**Table 1:** Antibiotic susceptibility of *Stenotrophomonas maltophilia* KJ and its derived strains.

Strain	MIC (μg/ml)			
	CAZ	LVX	SXT	MIN
KJ(pRK415)	>256	0.25	0.38	3
KJ(pHemI)	>256	0.125	0.25	3
KJ2(pRK415)	0.19	ND	ND	ND
KJ2(pHemI)	0.25	ND	ND	ND

MIC, minimum inhibitory concentration; ND, not determined; CAZ, ceftazidime; LVX, levofloxacin; SXT, trimethoprim–sulfamethoxazole; MIN, minocycline

### 3.5 Iron-depleted/hemin-available conditions modulate the antibiotic susceptibility of clinical isolates

Because *hemI* is fully activated under iron-depleted and hemin-available conditions (**Figure 4C**), conditions resembling infection niches, we asked whether such conditions might affect the antibiotic susceptibility of clinical *S. maltophilia* isolates. A total of 20 ceftazidime-susceptible clinical isolates plus KJ (or KJ2) were subjected to antibiotic susceptibility testing on Mueller–Hinton (MH) agar with or without DIP + hemin. All 21 strains (100%) showed increased MICs for ceftazidime and minocycline in the DIP + hemin medium. In contrast, the MIC responses of levofloxacin and trimethoprim/sulfamethoxazole were heterogeneous—increasing, decreasing, or unchanged (**Table 2**). Focusing on the strains that shifted from susceptible in MH to resistant in MH + DIP + hemin, we found that 71.4% (15/21), 10% (1/10), 0% (0/18), and 90% (9/10) lost susceptibility to ceftazidime, levofloxacin, trimethoprim/sulfamethoxazole, and minocycline, respectively (**Table 2**).

**Table 2:** Impact of iron-depleted and hemin availability on the antibiotic susceptibility of clinical *Stenotrophomonas maltophilia* isolates.

Isolate	MIC (μg/ml)							
	CAZ		LVX		SXT		MIN	
	None	D+H^a^	None	D+H^a^	None	D+H^a^	None	D+H^a^
KJ	ND	ND	0.25	0.064	0.38	0.047	3	12
KJ2	0.19	0.38	ND	ND	ND	ND	ND	ND
YT13	0.5	0.75	0.25	0.25	0.19	0.125	0.38	2
YT17	4	24	3	3	0.38	0.5	0.5	12
YT25	0.5	128	4	8	>32	>32	1	12
YT27	8	16	4	6	0.19	0.19	4	16
YT29	3	12	2	0.5	0.5	0.75	2	8
YT35	0.5	16	8	8	>32	>32	1	4
YT42	1.0	6	4	8	0.75	0.19	6	16
YT44	1.5	2	0.5	0.5	0.25	0.125	0.19	1.5
YT61	2	24	1.5	1.5	0.38	0.38	15	12
YT62	3	12	12	1.5	1.5	0.5	0.75	1.0
YT67	4	12	2	2	0.25	0.38	2	8
YT70	0.5	16	6	12	>32	>32	0.75	6
YT76	1.0	16	8	6	0.5	0.5	6	16
YT77	2	96	3	8	0.38	0.38	4	12
YT84	1.5	6	8	4	0.75	0.19	4	12
YT112	4	12	4	2	0.5	0.5	0.38	1.5
YT118	8	48	0.38	0.75	0.19	0.19	0.25	1.5
YT119	1.5	6	2	2	0.25	0.19	0.75	8
YT186	8	12	6	6	0.25	0.5	2	4
YT143	8	32	1	0.75	0.19	0.5	1.5	4

According to the guidelines of the Clinical and Laboratory Standards Institute (CLSI), the susceptibility breakpoints for CAZ, LVX, SXT, and MIN are ≤8, ≤2, ≤2, and ≤1, respectively. Gray shading denotes that the MIC is higher than the susceptibility breakpoint.

MIC, minimal inhibition concentration; ND, not determined; CAZ, ceftazidime; LVX, levofloxacin; SXT, trimethoprim–sulfamethoxazole; MIN, minocycline

a D+H: 2,2′-Dipyridyl (DIP) (30 μg/ml) and hemin (150 μM)

## 4 Discussion

Fur is the master repressor of iron acquisition genes under iron-replete conditions, and these genes are derepressed when iron is limited. In addition to Fur, many bacteria also encode secondary Fur-dependent systems that fine-tune gene expression in specific contexts. We previously identified several such regulators in *S. maltophilia*: AmpR (Liao et al., 2022), HemP (Shih et al., 2022), and the two-component SbiRS system (Wu et al., 2022). Here, we characterized a novel ECF sigma factor, i.e., HemI, that governs hemin acquisition. Although part of a *fecIRA*-like operon, *hemI*–*hemR*–*hemA_D_* is atypical: HemA_D_ is a truncated TBDT, and HemI is not autoregulated. In canonical SSC systems, the iron-liganded TBDT transduces a signal that releases the sigma factor from its anti-sigma factor (e.g., HemR) to activate transcription. Because HemA_D_ in KJ is defective, such signaling cannot occur; thus, HemR likely fails to function as an anti-sigma factor under iron-replete conditions, explaining the absence of autoregulation.

**Figure 6:**
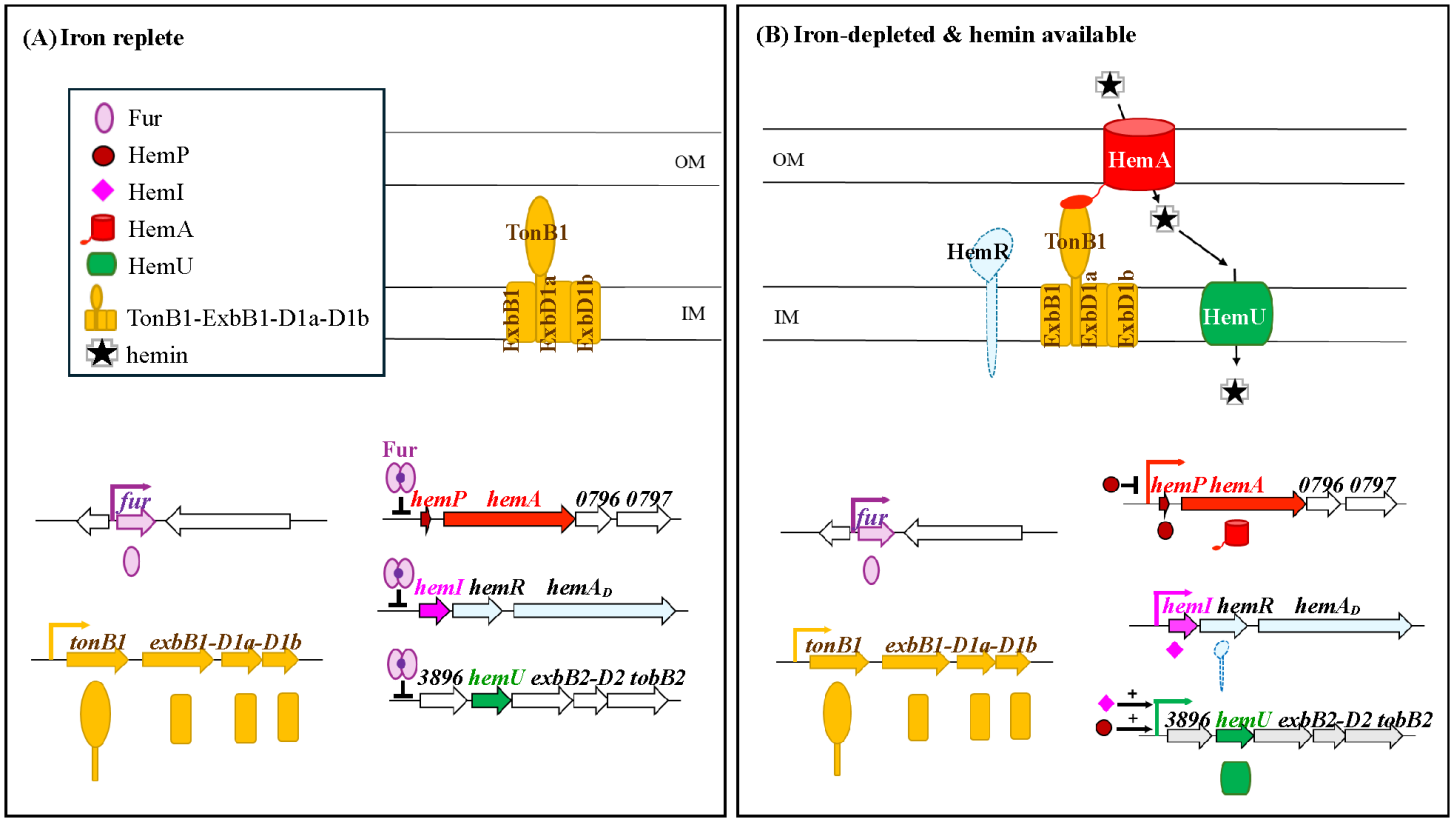
Proposed hierarchical model for the regulation of hemin acquisition in *Stenotrophomonas maltophilia*. **(A)** Under iron-replete conditions, Fur represses the *hemP*–*hemA*–*smlt0796*–*smlt0797*, *smlt3896*–*hemU*–*exbB2*–*exbD2*–*tonB2*, and *hemI*–*hemR*–*hemA_D_* operons. **(B)** Under iron-depleted conditions, Fur repression is relieved, allowing the expression of these operons. HemP negatively regulates *hemP*–*hemA*–*smlt0796*–*smlt0797*, while HemP and HemI are both required for the activation of *smlt3896*–*hemU*–*exbB2*–*exbD2*–*tonB2* in the presence of hemin.

Hemin acquisition genes are dispersed across multiple operons, but must be coordinately expressed. Integrating this and previous work (Liao et al., 2024; Shih et al., 2022), we propose a regulatory hierarchy (**Figure 6**): under iron-replete conditions, Fur represses *hemP*–*hemA*–*smlt0796*–*smlt0797*, *smlt3896*–*hemU*–*exbB2*–*exbD2*–*tonB2*, and *hemI*–*hemR*–*hemA_D_* (**Figure 6A**). Under iron depletion, Fur dissociates, allowing these operons to be expressed; hemin further enhances *smlt3896*–*hemU*–*exbB2*–*exbD2*–*tonB2* and *hemI*–*hemR*–*hemA_D_*. HemP negatively regulates *hemP*–*hemA*–*smlt0796*–*smlt0797*, while both HemP and HemI are essential for the activation of *smlt3896*–*hemU*–*exbB2*–*exbD2*–*tonB2* (**Figure 6B**).

Nutritional immunity is a common mechanism through which the host cells restrict pathogen viability. Because host tissues are typically iron-depleted but rich in hemin, pathogens must activate their hemin uptake machinery to thrive. Fur, HemP, and HemI are known regulators involved in the hemin acquisition of *S. maltophilia* (Liao et al., 2024; Shih et al., 2022). It is reasonable to expect that these regulators are likely to control additional stress adaptation genes beyond hemin acquisition. This inference is supported by antibiotic susceptibility testing (AST). HemI overexpression or iron-depleted/hemin-available conditions made strain KJ more susceptible to levofloxacin and trimethoprim/sulfamethoxazole, but strain KJ2 less susceptible to ceftazidime (**Tables 1**, **2**). Although the MIC alteration tendency was consistent in HemI overexpression and iron-depleted/hemin-available conditions, it was more significant in the iron-depleted/hemin-available conditions (**Tables 1**, **2**), implying that other unidentified iron-depleted/hemin-available-responsive regulators also modulate drug susceptibility, in addition to HemI.

Finally, we note a clinical implication: the Clinical and Laboratory Standards Institute (CLSI) standard AST assays use nutrient-replete MH medium, which does not mimic infection site microenvironments. Our findings suggest that the actual in-host MICs for ceftazidime and minocycline may be higher than the AST values, potentially leading to an underestimation of the resistance risk and treatment failure. Conversely, trimethoprim/sulfamethoxazole susceptibility was largely unaffected.

## Data Availability

The datasets presented in this study can be found in online repositories. The names of the repository/repositories and accession number(s) can be found in the article/**Supplementary Material**.
