# Supplementary material for: Involvement of HemI, an ECF sigma factor, in hemin acquisition and antibiotic susceptibility in Stenotrophomonas maltophilia
Source: Front Cell Infect Microbiol. 2025 Dec 23;15:1722701. doi: 10.3389/fcimb.2025.1722701 (PMC12772443; doi:10.3389/fcimb.2025.1722701)
Supplement: Supplementary file 1 [file DataSheet1.pdf]

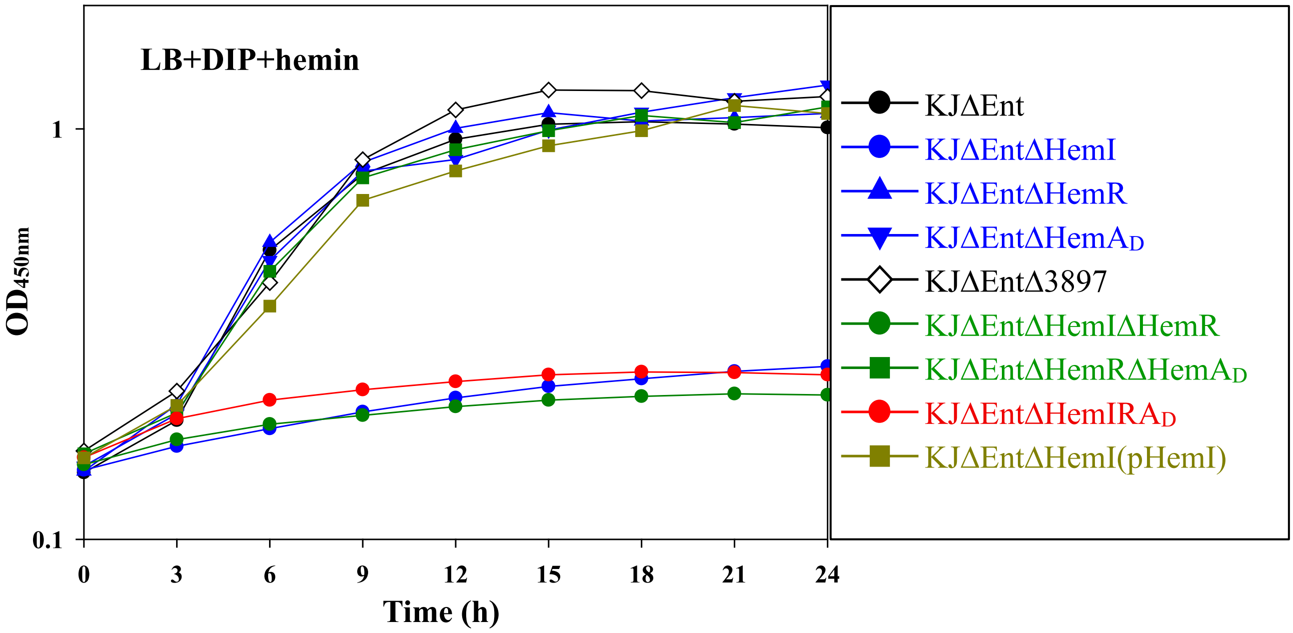

Fig. S1. Growth curve of parental strain KJΔEnt and its derived mutants grown under iron-depleted with hemin as sole iron source. An overnight bacterial culture was inoculated to LB broth supplemented with 50 μg/mL DIP and 150 μM hemin at an initial OD_{450} nm of 0.15. Bacterial growth was monitored by recording the OD_{450} nm for 24 h at intervals of 3 h. Graph is representative of at least three independent experiments.

(A)

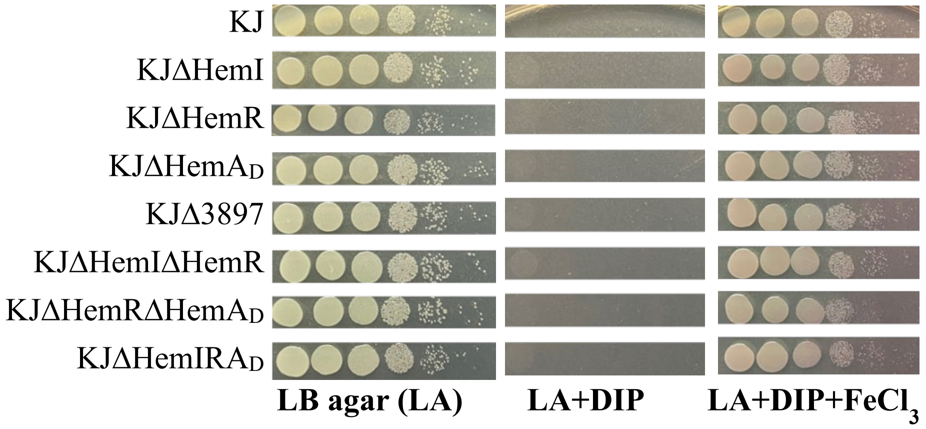

(B)

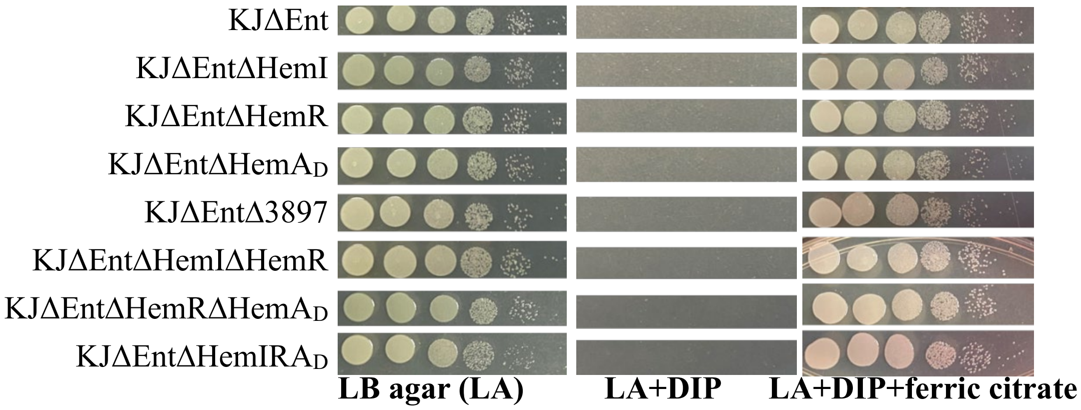

Fig. S2. Role of *smlt3897* and the *hemI-hemR-hemA$_D$* operon in the acquisition of ferric chloride and ferric citrate under iron-depleted conditions. Overnight cultures were inoculated into fresh LB broth at an initial OD_{450} = 0.15 and incubated for 5 h at 37°C. Cultures were then adjusted to 2 × 10^5 CFU/µL and subjected to 10-fold serial dilution. Five-microliter aliquots were spotted onto LB agar with or without the indicated supplements. Cell viability was assessed after 24 h at 37°C. Images are representative of at least three independent experiments. DIP, 2,2'-dipyridyl (50 µg/mL); $FeCl_3$, 35 µM; ferric citrate, 110 µM. (A) Role of *smlt3897* and *hemI-hemR-hemA$_D$* in ferric chloride utilization under iron-depleted conditions. (B) Role of *smlt3897* and *hemI-hemR-hemA$_D$* in ferric citrate utilization under iron-depleted conditions.

(A)

```
K279a, sm1t3898   1   ATGACGCCGCGCCTGCGGCACCGATTACCTGACAGGTTCCCCATG   45
                      ||||||||||||| ||||||| |||||||||||||||||||||||
KJ, hemAD         1   ATGACGCCGCGCCCGCGGCACTGATTACCTGACAGGTTCCCCATG   45
```

(B)

```
K279a, Sm1t3898   1   MTPRLRHRLPDRFPM   15

KJ, HemAD         1   MTPRPRH-LPDRFPM   15
```

Fig. S3. Alignment of the 1-45 nucleotide sequences and their encoded peptide sequences between *smlt3898* of strain K279a and *hemA$_D$* of strain KJ. The stop codon in *hemA$_D$* is highlighted in gray. (A) Alignment of the 1-45 nucleotides between *smlt3898* (K279a) and *hemA$_D$* (KJ). (B) Alignment of the first 15 amino-acid residues between *smlt3898* (K279a) and *hemA$_D$* (KJ).

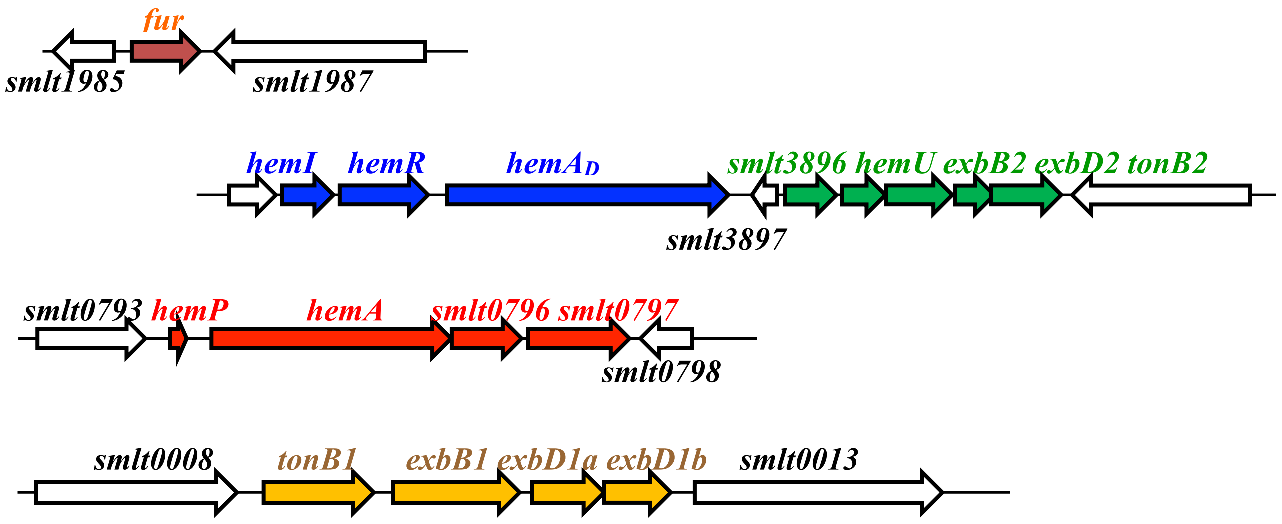

Fig. S4. Genomic organization of *fur*, *hemI-hemR-hemA$_D$*, *smlt3896-hemU-exbB2-exbD2-tonB2*, *hemP-hemA-smlt0796-smlt0797*, and *tonB1-exbB1-exbD1a-exbD1b* operons of *S. maltophilia*. Gene orientation is indicated by arrows. Genes belonging to the same operon are shown in the same color.

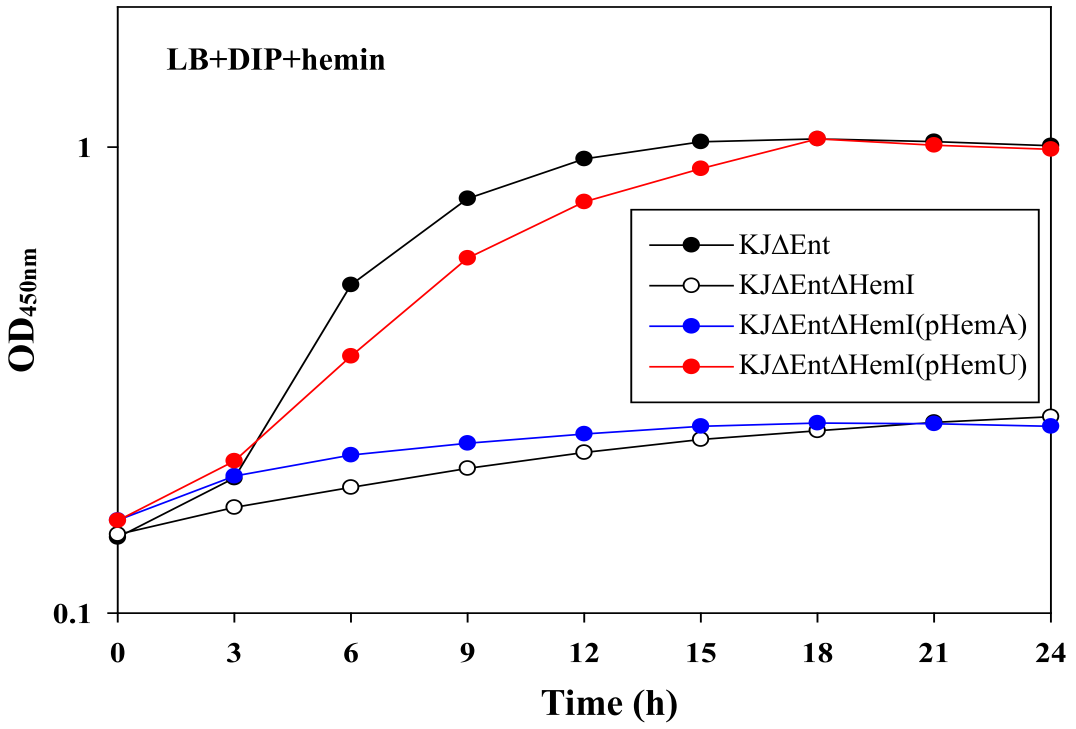

Fig. S5. Growth curve of parental strain KJΔEnt and its derived mutants grown under iron-depleted with hemin as sole iron source. An overnight bacterial culture was inoculated to LB broth supplemented with 50 μg/mL DIP and 150 μM hemin at an initial OD_{450} nm of 0.15. Bacterial growth was monitored by recording the OD_{450} nm for 24 h at intervals of 3 h. Graph is representative of at least three independent experiments.

(A)

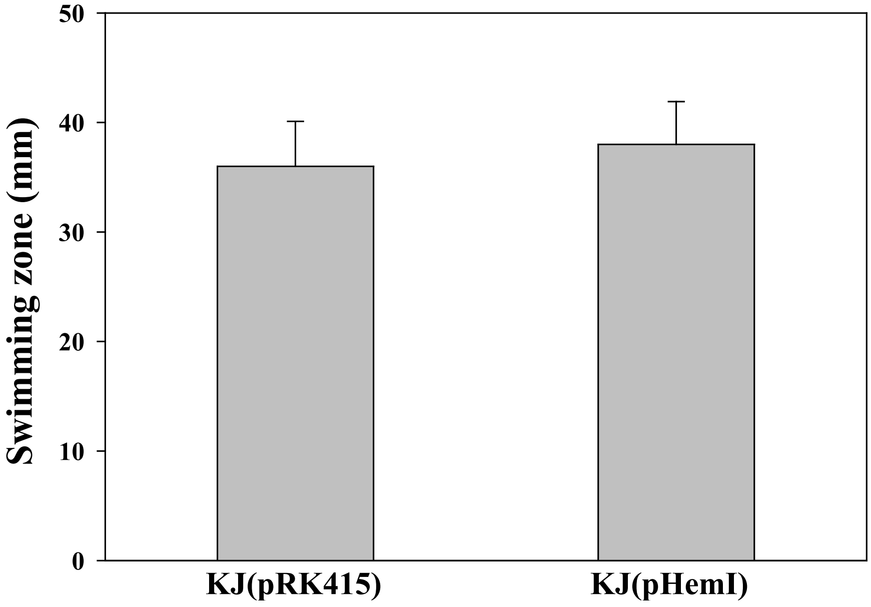

(B)

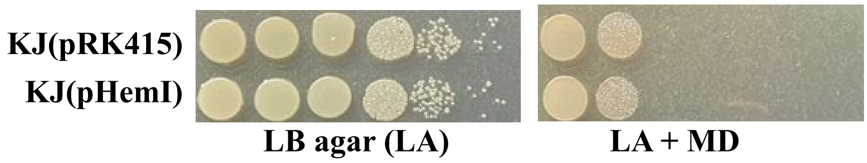

Fig. S6. Roles of HemI in swimming motility and menadione tolerance. (A) Swimming motility: overnight cultures were inoculated into swimming agar and incubated for 48 h at 37 °C. Swimming zones were recorded. Bars show the means of three independent experiments. Significance determined by Student's *t*-test. (B) Menadione tolerance: bacterial suspensions (2×10^5 CFU/µL) were serially diluted tenfold, and 5-µL aliquots were spotted onto LB agar without or with 60 µg/mL MD. Growth was assessed after 24 h at 37°C.

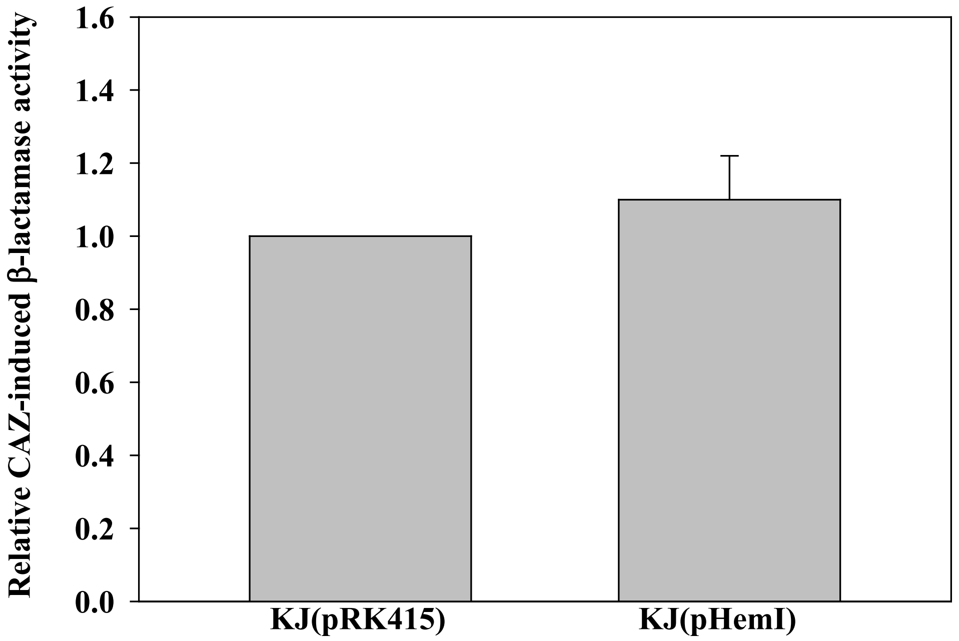

Fig. S7. Impact of HemI on CAZ-induced β-lactamase **activity**. Bacterial cells were grown overnight and reinoculated into 5 mL of fresh LB broth at an initial OD_{450} = 0.15. After 3 h, 50 µg/mL ceftazidime was added and cultures were incubated for an additional 30 min. Intracellular β-lactamase activity was assayed using nitrocefin as the substrate. One unit (Un) of activity was defined as the amount of enzyme hydrolyzing 1 nmol of nitrocefin per min. Specific activity (Un/mg) is expressed as nmol of nitrocefin hydrolyzed per min per mg of protein. Relative β-lactamase activity was normalized to the wild-type KJ level (set as 1). Bars show the means of three independent experiments. Error bars indicate standard errors of the mean.

Table S1 Bacterial strains and plasmids used in this study

Strain or plasmid	Genotype or properties	Reference
S. maltophilia		
KJ	A clinical *S. maltophilia* isolate	1
KJΔEnt	*S. maltophilia* KJ mutant of *entF* gene; nucleotides 52 to 3552 of *entF* gene deleted	2
KJΔEntΔ3897	*S. maltophilia* KJ mutant of *entF* and *smlt3897* genes; nucleotides 52 to 3552 of *entF* gene deleted, nucleotides 43 to 174 of *smlt3897* gene deleted	This study
KJΔEntΔHemI	*S. maltophilia* KJ mutant of *entF* and *hemI* genes; nucleotides 52 to 3552 of *entF* gene deleted, nucleotides 91 to 363 of *hemI* gene deleted	This study
KJΔEntΔHemR	*S. maltophilia* KJ mutant of *entF* and *hemR* genes; nucleotides 52 to 3552 of *entF* gene deleted, nucleotides 283 to 918 of *hemR* gene deleted	This study
KJΔEntΔHemA$_D$	*S. maltophilia* KJ mutant of *entF* and *hemA$_D$* genes; nucleotides 52 to 3553 of *entF* gene deleted, nucleotides 460 to 2418 of *smlt3898* gene deleted	This study
KJΔEntΔHemI ΔHemR	*S. maltophilia* KJ mutant of *entF, hemI,* and *hemR* genes; nucleotides 52 to 3552 of *entF* gene deleted, nucleotides 91 to 363 of *hemI* gene deleted, nucleotides 283 to 918 of *hemR* gene deleted	This study
KJΔEntΔHemR ΔHemA$_D$	*S. maltophilia* KJ mutant of *entF, hemR,* and *hemA$_D$* genes; nucleotides 52 to 3552 of *entF* gene deleted, nucleotides 283 to 918 of *hemR* gene deleted, nucleotides 460 to 2418 of *smlt3898* gene deleted	This study
KJΔEntΔHemIRA$_D$	*S. maltophilia* KJ mutant of *entF, hemI, hemR,* and *hemA$_D$* genes; nucleotides 52 to 2552 of *entF* gene deleted, nucleotides 91 to 363 of *hemI* gene deleted, nucleotides 283 to 918 of *hemR* gene deleted, nucleotides 460 to 2418 of *smlt3898* gene deleted	This study
KJΔFur	*S. maltophilia* KJ mutant of *fur* gene; nucleotides 19 to 348 of *fur* gene deleted	2
KJΔHemP	*S. maltophilia* KJ mutant of *hemP* gene; nucleotides 31 to 171 of *hemP* gene deleted	3

KJΔHemI	*S. maltophilia* KJ mutant of *hemI* gene; nucleotides 91 to 363 of *hemI* gene deleted	This study
KJΔFurΔHemI	*S. maltophilia* KJ mutant of *fur* and *hemI* genes; nucleotides 19 to 348 of *fur* gene deleted, nucleotides 91 to 363 of *hemI* gene deleted	This study
KJΔFurΔHemR	*S. maltophilia* KJ mutant of *fur* and *hemR* genes; nucleotides 19 to 348 of *fur* gene deleted, nucleotides 283 to 918 of *hemR* gene deleted	This study
KJΔFurΔHemI ΔHemR	*S. maltophilia* KJ mutant of *fur, hemI,* and *hemR* genes; nucleotides 19 to 348 of *fur* gene deleted, nucleotides 91 to 363 of *hemI* gene deleted, nucleotides 283 to 918 of *hemR* gene deleted	This study
E. coli		
DH5α	F- φ80d/*acZΔM15 Δ*(*lacZYA-argF*)*U169 deoR recA1 endA1 hsdR17* (r_k^- m_k^+) *phoA supE44λ thi-1 gyrA96 relA1*	Invitrogen
S17-1	λpir^+ mating strain	4
**Plasmids**		
pEX18Tc	*sacB oriT*, Tc^r	5
pRK415	Mobilizable broad-host-range plasmid cloning vector, RK2 origin; Tc^r	6
pΔHemI	pEX18Tc with a *hemI* gene whose nucleotides 91 to 363 were deleted, Tc^r	This study
pΔHemR	pEX18Tc with a *hemR* gene whose nucleotides 283 to 918 were deleted, Tc^r	This study
pΔ$HemA_D$	pEX18Tc with a *smlt3898* gene whose nucleotides 460 to 2418 were deleted; Tc^r	This study
pHemA	pRK415 with an intact *hemA* gene and ; Tc^r	3
pHemU	pRK415 with an intact *hemU* gene; Tc^r	7
pHemI	pRK415 with an intact *hemI* gene; Tc^r	This study
$pHemP_{xylE}$	pRK415 with a P_{hemP}::*xylE* transcriptional fusion construct; Tc^r	3
$p3896_{xylE}$	pRK415 with a $P_{smlt3896}$::*xylE* transcriptional fusion construct; Tc^r	7
$pHemI_{xylE}$	pRK415 with a P_{hemI}::*xylE* transcriptional fusion construct; Tc^r	This study
$pTonB1_{xylE}$	pRK415 with a P_{tonB1}::*xylE* transcriptional fusion construct; Tc^r	This study

1. Hu, R. M., Huang, K. J., Wu, L. T., Hsiao, Y. J., Yang, T. C. (2008) Induction of L1 and L2 β-lactamases of *Stenotrophomonas maltophilia*. *Antimicrob Agents Chemother.* 52(3), 1198-1200.
2. Liao, C. H., Chen, W. C., Li, L. H., Lin, Y. T., Pan, Z. Y., Yang, T. C. (2020) AmpR of *Stenotrophomonas maltophilia* is involved in stenobactin synthesis and enhanced β-lactam resistance in an iron-depleted condition. *J Antimicrob Chemother.* 75, 3544-3551.
3. Shih, Y. L., Wu, C. M., Lu, H. F., Li, L. H., Lin, Y. T., Yang, T. C. (2022) Involvement of the *hemP-hemA-smlt0796-smlt0797* operon in hemin acquisition by *Stenotrophomonas maltophilia*. *Microbiol Spectr.* 10(3), e0032122.
4. Simon, R., O'Connell, M., Labes, M., Puhler, A. (1986) Plasmid vector for the genetic analysis and manipulation of *Rhizobia* and other Gram-negative bacteria. *Methods Enzymol.* 118, 640-659.
5. Hoang, T. T., Karkhoff-Schweizer, R. R., Kutchma, A. J., Schweizer, H. P. (1998) A broad-host-range Flp-FRT recombination system for site-specific excision of chromosomally-located DNA sequences: application for isolation of unmarked *Pseudomonas aeruginosa* mutants. *Gene.* 212, 77-86.
6. Keen, N. T., Tamaki, S., Kobayashi, D., Trollinger, D. (1998) Improved broad-host-range plasmids for DNA cloning in gram-negative bacteria. *Gene,* 70, 191-197.
7. Liao, C. H., Lu, H. F., Yang, C. W., Yeh, T. Y., Lin, Y. T., Yang, T. C. (2024) HemU and TonB1 contribute to hemin acquisition in *Stenotrophomonas maltophilia*. *Front Cell Infect Microbiol.* 14, 1380976.

Table S2 Primers used in this study

primer	Sequence (5'→3')	Amplified region	Purpose
HemIN-F<br>HemIN-R	CTGAGCTCATTTGCCGCCAGTGACC<br>TCGGTACCCAGGATGCGTTGCAC	285 bps upstream of *hemI* and nucleotides 1-90 of *hemI*	pΔHemI construction
HemIC-F<br>HemIC-R	CCAGGTACCGGCTCAACCGCCT<br>GTCTCTAGAGGCCAGCCACG	nucleotides 364-501 of *hemI* and 194 bps downstream of *hemI*	
HemR-F<br>HemR-R	GCAAAGCTTGACCCAGGCCGAGAT<br>CGGTCTAGAGTGGCCGGATACACC	195 bps upstream of *hemR*, *hemR*, and 432 bp downstream of *hemR*	pΔHemR construction
$HemA_D$-F<br>$HemA_D$-R	GCCAAGCTTGGATGATGTTCCCGAT<br>CACGAATTCGCGACCGATCAGTACT	153 bps upstream of *smlt3898*, *smlt3898*, and 17 bp downstream of *smlt3898*	p$\Delta HemA_D$ construction
3897-F<br>3897-R	GCGTCTAGAGCAGGGTTGGCATACA<br>TCAGGAATTCGGACAAGGACTGGAA	307 bps upstream of *smlt3897*, *smlt3897*, and 377 bp downstream of *smlt3897*	pΔ3897 construction
HemI-F<br>HemI-R	TGCAAGCTTTGATGCGAACGTGACT<br>ACCGAATTCACGGTCCAGTGATCAA	84 bps upstream of *hemI*, *hemI*, and 61 bp downstream of *hemI*	pHemI construction
HemIN-F<br>HemIN-R	CTGAGCTCATTTGCCGCCAGTGACC<br>TCGGTACCCAGGATGCGTTGCAC	285 bps upstream of *hemI* and nucleotides 1-89 of *hemI*	$pHemI_{xylE}$ construction
TonB1N-F<br>TonB1N-R	CAGGTACCAGCCGGACTACCA<br>CTTCTAGAGGGTTGTTCGTA	396 bps upstream of *tonB1* and nucleotides 1-48 of *tonB1*	$pTonB1_{xylE}$ construction
HemAQ93-F<br>HemAQ93-R	CCTGCTCAGCAAACTGGTCT<br>AGCACATTGGTATCGGTGGT	nucleotides 705 to 797 of *hemA* gene	qRT-PCR
HemUQ100-F<br>HemUQ100-R	ATGCGCTGGCTCTGGTTC<br>CCAGGATCAGGAAGATGGTG	nucleotides 1 to 100 of *hemU* gene	qRT-PCR
HemIQ110-F<br>HemIQ110-R	GACAATCCGCGTGCCTACC<br>GCTCCCCACAGGTAGTGGT	nucleotides 160 to 269 of *hemI* gene	qRT-PCR
TonB1Q100-F<br>TonB1Q100-R	CGGTACGAACAACCCGATGA<br>TGAGGAGCATCATGAAGGCG	nucleotides 25 to 124 of *tonB1* gene	qRT-PCR
16S rDNA-F<br>16S rDNA-R	GACCTTGCGCGATTGAATG<br>CGGATCGTCGCCTTGGT	nucleotides 211 to 286 of 16S rDNA	qRT-PCR
